# 

**Published:** 1855-05

**Authors:** 


### CONTENTS

ORIGINAL COMMUNICATIONS:— PAGE

ART. I.—Report of a Case of Puerperal Convulsions, with Remarks on the Use of Chloroform. By William Clarke, M.D. . . . . 193

ART. II.—Monthly Report on the Sanitary Condition of Chicago and the Progress of Epidemics. By N. S. Davis, M.D. . . . . 198

ART. III.—Uterine Polypus. By George Higgins, M.D. . . . . 205

ART. IV.—Hints to Young Practitioners on the Application of Certain Bandages, &c. By B. E. Dodson, M.D. . . . . . . 207

SELECTIONS:

Case of Gastrotomy for the Removal of a Leaden Bar . . . . . 208

Sedative Powers of the Root of Yellow Jessamine . . . . . 211

Ulceration of the Legs . . . . . . . . . . . 214

BOOK NOTICES, . . . . . . . . . . 221

EDITORIAL:

Communication from H. A. J. New Orleans . . . . . . 228

Advertising and the New York University . . . . . . . 232

The Medical Department of the University of Michigan . . . 234

Miscellaneous Items . . . . . . . . . . . 236

